# Perspectives on the measurement of self‐perceived cognitive function in older adults

**DOI:** 10.1002/dad2.70158

**Published:** 2025-11-06

**Authors:** Elke Butterbrod, Laura Rabin, Douglas Tommet, Richard N. Jones, Mark A. Dubbelman, Paul K. Crane, Frank Jessen, Wiesje M. van der Flier, Katherine A. Gifford, Sietske A. M. Sikkes, ISTAART SCD PIA, and the Subjective Cognitive Decline Initiative (SCD‐I) Working Group

**Affiliations:** ^1^ Department of Clinical Neuro‐ and Developmental Psychology Vrije Universiteit Amsterdam Amsterdam the Netherlands; ^2^ Department of Neurology Alzheimer Center Amsterdam University Medical Center Amsterdam the Netherlands; ^3^ Department of Psychology Brooklyn College and Graduate Center of The City University of New York Brooklyn New York USA; ^4^ Saul R. Korey Department of Neurology Albert Einstein College of Medicine New York New York USA; ^5^ Department of Neurology Warren Alpert Medical School of Brown University Providence Rhode Island USA; ^6^ Department of Neurology Center for Alzheimer Research and Treatment Brigham and Women's Hospital Boston Massachusetts USA; ^7^ Department of Neurology Massachusetts General Hospital Harvard Medical School Boston Massachusetts USA; ^8^ Department of Medicine University of Washington Seattle Washington USA; ^9^ Department of Psychiatry University of Cologne Medical Faculty Köln Germany; ^10^ German Center for Neurodegenerative Diseases (DZNE) Bonn Germany; ^11^ Department of Epidemiology and Data Science Amsterdam University Medical Center Amsterdam The Netherlands; ^12^ Department of Anatomy & Neurobiology Boston University School of Medicine Boston Massachusetts USA; ^13^ Department of Neurology Vanderbilt University Medical Center Nashville Tennessee USA

**Keywords:** Alzheimer's disease, cognitive function, preclinical Alzheimer's disease, subjective cognitive decline, survey

## Abstract

**INTRODUCTION:**

This survey investigated perspectives of research and clinical professionals on optimal content and features of measurement of self‐perceived cognitive functioning.

**METHODS:**

Respondents were professionals working with older adults with self‐reported cognitive concerns. The survey addressed views on harmonization and preferences for items, response formatting, practical features, and instrument validation. We evaluated item preferences in consideration of a previous statistical harmonization.

**RESULTS:**

Ninety professionals from 20 different countries completed the survey. Most professionals (87%) indicated a need for a harmonized instrument. Respondents agreed that an instrument should measure current ability alongside change therein, focus on memory, and adopt Likert scale responses. Recommendations for assessment timeframe, practical features, and validation priorities varied. Respondents differentially endorsed items previously found to be statistically informative.

**DISCUSSION:**

Respondents agreed on overarching measurement topics, with varying recommendations for specific content and features. Together with statistical information, these results provide a starting point for a harmonized instrument.

**Highlights:**

- Professionals see a need for a harmonized tool to measure cognitive concerns.
- Professionals have diverse preferences for measurement content and its validation.
- Item relevance as seen by professionals aligned considerably with statistical value.
- Integration of statistical information with expert and patient opinion is crucial.

## 1 INTRODUCTION

Interest in the potential relevance of subjective cognitive decline (SCD) in the context of early detection of Alzheimer's disease (AD) is growing.^1^, ^2^, ^3^, ^4^ SCD is characterized by a self‐experienced persistent decline in cognitive capacity compared to a previously normal status in the absence of objective impairment on neuropsychological tests.^1^, ^5^ Although the perceived decline in cognition is largely unspecific to any particular neurological disorder,^1^ SCD can occur as an early symptomatic stage of AD in some individuals.^2^, ^6^ Several features of the experienced decline, such as onset within the last 5 years, concerns pertaining to memory in particular, and worry associated with the experienced decline, are referred to as SCD *plus* features.^1^ These concerns indicate a higher likelihood of AD pathology and future progression to mild cognitive impairment (MCI) or dementia,^5^, ^7^ in particular if they occur in combination with each other and other SCD *plus* features that pertain to the individual, such as *APOE* E4 carriership and age at onset over 60.^1^, ^8^, ^9^, ^10^

Systematic assessment of self‐perceived cognitive functioning can serve as a non‐invasive, low‐cost tool to facilitate the detection of SCD and potentially improve the ability to target individuals for clinical trials in an early disease phase.^11^ Despite the widely acknowledged relevance, several challenges still hamper implementation of self‐perceived cognitive functioning assessment. A key issue concerns the heterogeneity that exists in objectives, samples, and instruments used to study the construct,^12^, ^13^ which limits the interpretability and generalizability of findings. Most existing questionnaires also do not include all, if any, complaint features of SCD *plus*, thereby potentially missing highly relevant information. Finally, professionals can have different purposes for and needs when measuring self‐perceived cognitive function, and such (differences in) considerations should be more thoroughly understood.

Adequate measurement is central to advancements in diagnosis, prognosis, and intervention.^14^ The Subjective Cognitive Decline Initiative (SCD‐I) Working Group, part of the International Society To Advance Alzheimer's Research and Treatment (ISTAART) SCD Professional Interest Area (PIA), has been engaged in ongoing efforts to facilitate the development of a harmonized instrument consisting of items with the highest information value that can measure across different levels of self‐perceived cognitive functioning (from poor to good functioning) and allow for comparison across different settings, with characteristics that ensure feasibility and acceptability for a variety of users.^13^ To this end, the working group has mapped the heterogeneity of existing assessment methods^13^ and provided recommendations for operationalizing SCD criteria in research.^6^ In a study by Rabin et al. (2023), over 600 existing items from commonly used questionnaires were harmonized onto a single trait of self‐perceived cognitive functioning (referred to as the subjective cognition factor) using item response theory (IRT) modeling.^15^ The information value of the items at different levels of functioning was also established.^15^, ^16^

**RESEARCH IN CONTEXT**

1. **Systematic review**: Despite its relevance, significant heterogeneity in measurement tools hinders widespread assessment of self‐perceived cognitive functioning. The Subjective Cognitive Decline Initiative (SCD‐I) Working Group has been working to harmonize assessment and develop an instrument that spans a range of functional levels to improve measurement feasibility and acceptability across diverse settings.
2. **Interpretation**: In our survey, professionals who work with SCD populations expressed several clear preferences for how cognitive concerns should be captured. Respondents differentially endorsed items previously found to be statistically informative, and variability in recommendations for practical features and validation was observed.
3. **Future directions**: Input from older adults with cognitive concerns and informants as a next step is imperative. Integration of diverse expert opinion, participant preferences, and statistical results can serve as a basis for instrument development. A harmonized instrument could ultimately consist of a core set of informative items supplemented with flexible modules to suit measurement goals.

While sophisticated statistical models like IRT provide a rich source of psychometric information,^17^ expert opinion of professionals is imperative for developing and improving instruments.^14^, ^18^ It allows us to compare and supplement psychometric information with experience‐based perspectives. Furthermore, considering users’ preferences for practical features and validation priorities of an instrument is essential for implementation. The current study surveyed professionals involved in care and research of self‐perceived cognitive functioning in older adults about their perspectives on essential features of and recommendations for its measurement.

## 2 METHODS

### 2.1 Survey sample and administration

This study is a cross‐sectional, international survey. We targeted professionals working with older adults with SCD through research and/or clinical practice. The survey was distributed through the ISTAART SCD PIA, the International Neuropsychological Society's Dementia Special Interest Group, the Dutch Society for Neuropsychology, and the authors’ professional networks that included professionals involved in research about SCD worldwide. Responses were recorded from July 2022 through November 2022.

The survey was programmed with the Qualtrics platform and, in accordance with the General Data Protection Regulation guidelines, used an anonymous link, ensuring that no personal information (such as IP or e‐mail addresses) was gathered. Respondents provided informed consent before starting the survey and were not required to answer any given question in order to proceed forward through the questionnaire. Unsubmitted surveys were not processed and were automatically discarded after 20 days. The protocol for this survey was evaluated by the Medical Ethical Committee of Amsterdam University Medical Center.

### 2.2 Survey content

The survey consisted of four parts (Supporting Information) and involved a combination of multiple‐choice questions, open‐ended questions, allocation of percentages, and two‐item selections.

1. **Part I** (*Background and practices*): Information was gathered about respondents’ gender, educational level, professional setting (e.g., hospital, university, rehabilitation center), discipline (e.g., psychology, medicine, including subdomains), profession (e.g., clinician, researcher, instructor), and years of experience. Respondents were also asked to indicate whether they routinely used instruments to assess self‐perceived cognitive functioning in older adults and to what degree they perceived a need for a harmonized instrument. These latter two items were multiple choice with an option to elaborate in a text box.
2. **Part II** (*Item content*): Perspectives on the relevance of item content were explored. Respondents were shown two separate lists of item stems (one list of 20 memory item stems and one list of 30 non‐memory item stems) taken from items that showed relatively high information values, as found by Rabin et al. (2023). The memory versus non‐memory distinction was made because memory items alone made up over 50% of the original item bank (15). Respondents were asked to select up to eight items from each list that they viewed as most relevant for the measurement of self‐perceived cognitive functioning in older adults. We compared the item ranking (from most chosen to least chosen) to the previously reported item information values. Subsequently, respondents were shown a list of various cognitive domains: memory, attention/working memory/processing speed, language, executive function, basic calculation and arithmetic tasks, orientation (to person, time, place, or situation), and visuospatial skills (for details on domains, see Rabin et al.^13^). Respondents were asked to allocate a percentage to each domain based on how they would ideally cover each domain on a questionnaire. They were able to write in a maximum of two additional domains that they deemed relevant but that were not included in the list. The total allocated percentage had to add up to 100%.
3. **Part III** (*Item format*): Perspectives on item features were assessed, including the most relevant timeframe to measure, measuring ability and/or change, and optimal response formats.
4. **Part IV** (*Questionnaire features*): Respondents selected up to three preferences for practical features and approaches to validation. Practical features included, for example, brevity and cost. Validation approaches included, for example, the relationship with biomarkers and predictive value. Both lists were composed of 10 options. For both questions, respondents could write in additional preferences and elaborate on choices in a comment box.

### 2.3 Data analyses

Descriptive analyses were performed to characterize the sample and responses to the multiple‐choice questions and item content preferences. We additionally described preferences for items (see part II of the survey description) separately for clinical versus research professionals. We also investigated differences in preferences for practical features and validation approaches (see part IV of the survey description) between clinical versus research professionals with chi‐squared tests (α = 0.05). These were not the only professions in our sample, but they were the target sample, meaning they were likely to make up the overwhelming majority and the most likely users of an instrument. Differences between these groups in particular were deemed relevant. Open‐ended questions and additional comments respondents made to closed questions were described.

## 3 RESULTS

### 3.1 Respondent sample

As shown in Table 1, 90 respondents (66% female) from four continents (20 countries) submitted a response to the survey. Almost half of the 90 respondents had obtained a PhD/PsyD degree (46% of respondents) and reported over 10 years of experience in the field of SCD (44%). Thirty‐nine (43%) indicated routinely using an instrument to assess SCD in older individuals for clinical or research purposes. In total, 16 unique instruments were mentioned, of which nine were also included in the item bank of the IRT study^15^ (Table S1).

**TABLE 1:** Characteristics of respondent sample.

Characteristic	*N* (%)
Geographical location^b^ (*N* = 86)	
Europe	51 (59)
North America	23 (27)
South America	7 (8)
Asia	5 (6)
Highest degree^b^ (*N* = 86)	
M.Sc.	25 (29)
M.D.	16 (19)
PhD/PsyD	42 (46)
Other	3 (4)
Profession^a^ (*N* = 87)	
Clinician	44 (51)
Researcher	61 (70)
Other (e.g., instructor)	12 (14)
Professional setting^a^ (*N* = 87)	
Hospital	48 (55)
University	40 (46)
Care home	12 (14)
Other (e.g., rehab facility)	23 (26)
Discipline^a^ (*N* = 87)	
(Neuro‐)Psychology	51 (48)
Medicine	20 (23)
Gerontology	9 (10)
Other (e.g., epidemiology)	26 (24)
Professional experience^b^ (*N* = 86)	
Less than 5 years	27 (31)
5 to 10 years	16 (18)
More than 10 years	43 (49)
Routinely uses an instrument to assess SCD	39 (43)

^a^ Choice count shown, multiple options per respondent possible.

^b^ Not completed by all respondents.

A large majority (79/90, 87%) of respondents indicated seeing “somewhat” to a “strong” need for a self‐perceived cognitive functioning harmonized instrument. Professionals added reasons for the need in the open‐ended section, reporting that different instruments have been shown to measure different outcomes (*n* = 1) and that it would help facilitate comparability (*n* = 1). Two of the 79 respondents who saw a need for harmonization added that such a measure would specifically aid research. The minority that reported seeing “no need” for a harmonized measure (11/90, 13%) reasoned that existing instruments provide sufficient value but mostly require good clinical cut‐offs or that observation and clinical interview are sufficient to measure SCD in this population.

### 3.2 Item content and domain selection

**FIGURE 1:**
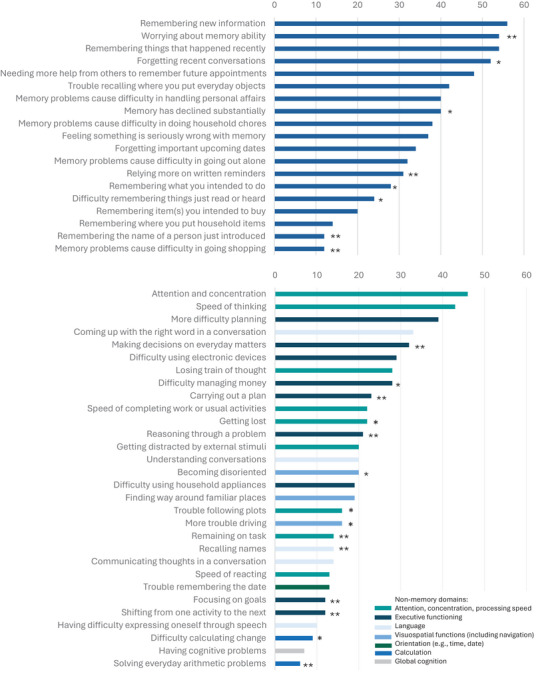
Ranking of memory and non‐memory items based on response percentage (i.e., percentage of respondents that chose the item as one of the most relevant), ordered from most commonly chosen to least commonly chosen. Items that were identified as “high information value” in the harmonization study by Rabin et al. (2023) are indicated: * showed high information value for worse self‐perceived cognitive functioning/high level of concerns (θ −1 to −2), ** showed high information value for better self‐perceived cognitive functioning/low level of concerns (θ 0 to 1).

Figure 1 shows the ranking of item stems in the memory and non‐memory domains. Items that were found to have high information value for different levels of self‐perceived cognition according to the statistical harmonization are indicated in this figure for reference. Items with high information value for the high functional level indicate that they measure particularly well for better levels of self‐perceived cognition (i.e., individuals with no problems), while items with high information value for the low functional level indicate they measure particularly well for poor self‐perceived cognition (i.e., individuals with substantial problems). Within the memory domain, items specifically referring to consolidation and/or retrieval of new material (new information, recent events, and conversations), planned future action, and worry about memory ability made up the top five chosen memory items (selected as relevant by 48% to 56% of respondents). For non‐memory items, attention and concentration, speed of thinking, more difficulty planning, coming up with the right words in conversation, and making decisions on everyday matters composed the top five (selected by 32% to 46% of respondents).

Figure S1 shows the item preferences of researchers and clinicians. The most popular memory item among researchers was “remembering things that happened recently” (70%) and for the non‐memory items “attention and concentration” (57%). The most popular items of clinicians were “remembering where you put household items,” “memory causing difficulty in going shopping” (both 50%), and “remaining on task” (53%). We observed that, among clinicians, most items were selected by 30% to 40% and the most popular ones by 50% of respondents. Among researchers, the least popular memory and non‐memory items were chosen by 4% and 0% and the most popular items by 70% and 57%, respectively.

When asked about the percentage coverage that should be assigned to different cognitive domains in a questionnaire, memory was the clear primary domain, with a mean of 32% desired coverage (Table 2). Almost all respondents allocated the coverage over multiple domains. Two respondents allocated the entire 100% to memory.

**TABLE 2:** Percentages allocated to cognitive domains by respondents.

Domain	Mean % ± SD
Memory	32 ± 18
Attention/working memory/processing speed	15 ± 8
Language	13 ± 8
Executive function	15 ± 8
Basic calculation and arithmetic tasks	6 ± 10
Orientation to person, time, place, or situation	9 ± 7
Visuospatial skills	8 ± 10
Social cognition (*n* = 7)^a^	7 ± 2
Emotional liability (*n* = 2)^a^	10 ± 0
Abstraction (*n* = 1)^a^	6 ± 0
Writing (*n* = 1)^a^	5 ± 0

*Note*: Mean percentages (± SD) allocated to each cognitive domain by respondents are shown.

^a^ Domain written in by respondent(s), ordered according to number of respondents (e.g., *n* = 7 means seven respondents added this domain).

### 3.3 Item format

**FIGURE 2:**
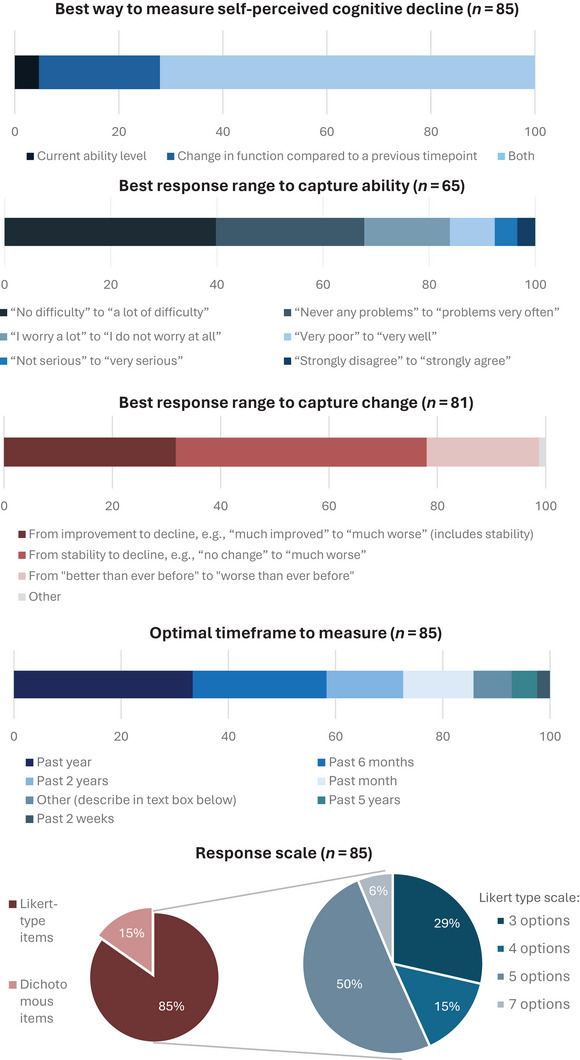
Professionals’ responses to item format questions (in percentage).

Figure 2 shows respondents’ choices with regard to item format. The majority (72%) indicated that a questionnaire should measure both current ability level and change compared to a previous time point, as opposed to either ability alone (5%) or change alone (23%). Regarding the response range for current ability level, the preferred range was the degree of *difficulty* an individual experiences when executing a task or using a function (chosen by 40%), while the least popular response range was agreement or disagreement with a statement (chosen by 3%). Respondents indicated that change should be measured from stability to decline (46%) rather than from improvement to decline. Respondents overwhelmingly preferred Likert‐type (85%) over dichotomous items (15%), with a five‐response option response scale selected most often (50%). Recommendations for the most relevant timeframe to measure were varied, with the past year (33%) and the past 6 months (25%) as the top two preferences. None of the respondents chose the past 10 years. One respondent mentioned that the timeframe could vary, for example, a longer timeframe at baseline or past year for follow‐ups.

### 3.4 Questionnaire features

Respondents’ preferences for practical and validation features are presented in Table 3. The most commonly chosen validation features were association with current performance on neuropsychological tests and the ability to dissociate cognitive concerns related to neurodegenerative cognitive decline from concerns related to other factors (both selected by 54% of respondents as one of their preferences). The chosen popular practical features were ease of administration (selected by 38%) and availability of an informant version (selected by 38%). The least chosen were clear administration guidelines (practical) and association with positron emission tomography (PET) biomarkers (validation), selected by 14% and 6% of respondents, respectively.

**TABLE 3:** Respondents’ preferences for validation and practical features.

Practical features	Choice count	Choice percentage (%)	Response percentage (%)
Ease of administration (little instruction needed)	33	13%	38%
Has a companion informant version/form	33	13%	38%
Availability of an appropriate normative comparison group	32	13%	37%
Brevity	29	12%	34%
Possibility to administer online (can be completed outside of appointments/clinic)	26	11%	30%
Has a cut‐off score for self‐perceived cognitive decline	19	8%	22%
Ease of scoring	13	5%	15%
Cost	12	5%	14%
Clear administration guidelines	12	5%	14%
Other (self‐written feature)*	3	1%	3%
Total (*respondent n =* 86)	*247*	*100%*	
Validation features	Choice count	Choice %	Response %
Association with current performance on neuropsychological tests	47	20%	54%
Able to dissociate cognitive concerns related to neurodegenerative cognitive decline from concerns related to other factors (e.g., mood, personality, medical variables)	47	20%	54%
Predictive value for clinical diagnosis (of MCI or dementia)	35	15%	40%
Predictive value for future decline in performance on neuropsychological tests	26	11%	30%
Predictive value for future decline in daily functioning (e.g., Instrumental Activities of Daily Living)	26	11%	30%
Sensitivity to changes in self‐perceived cognitive functioning over time (responsiveness)	26	11%	30%
Association with plasma biomarkers of Alzheimer's disease	9	4%	10%
Association with biomarkers of dementia derived from MRI	7	3%	8%
Association with CSF biomarkers of dementia	6	3%	7%
Association with biomarkers derived from PET	5	2%	6%
Other (self‐written feature)*	1	0%	1%
Total (*respondent n = 87*)	*235*	*100%*	

Respondents indicated a maximum of three preferences. Choice count = # of times the feature was chosen, choice percentage = percentage of total choices made that included this feature, response percentage = % of respondents who chose this feature as a preferred feature.

Abbreviations: CSF, cerebrospinal fluid; MCI, mild cognitive impairment; MRI, magnetic resonance imaging; PET, positron emission tomography.

* Written in answers for practical feature: test validity, informative value for self‐awareness. No write‐in answer was provided for validation.

### 3.5 Comparison of preferences for items and questionnaire features of clinicians versus researchers

In the selection of most relevant items, we found that higher proportions of researchers (compared to non‐researchers) selected remembering new information (61%) and relying more on written reminders (44%), and lower proportions selected remembering where you put household items (4%) and communicating thoughts in a conversation (9%) (*p* < 0.05). Higher proportions of clinicians (compared to non‐clinicians) selected items related to memory problems causing difficulty in going out alone (49%), remembering the name of a person just introduced (17%), and remaining on task (53%), while lower proportions selected items related to making decisions on everyday matters (43%) and forgetting recent conversations (43%) (*p* < 0.05). Figure S1 presents item selections stratified for clinicians and researchers.

Several preferences for practical features of a questionnaire were different between respondents who indicated being primarily researchers or clinicians. For the validation features, predictive value of the questionnaire for future decline in performance on neuropsychological tests and responsiveness (i.e., sensitivity to changes over time) were chosen by higher proportions of researchers (both features 38%) than non‐researchers (both features 10%) (*p* < 0.01). Association with current performance on neuropsychological tests and predictive value for future decline in daily functioning were chosen by higher proportions of clinicians (68% and 43%) compared to non‐clinicians (37% and 15%) (*p* < 0.01). In terms of practical features, the possibility to administer online was chosen by a higher proportion of researchers (34%) than non‐researchers (14%) (*p *= 0.041). A higher proportion of clinicians chose clear administration guidelines (20%) and availability of a normative comparison group (48%) compared to non‐clinicians, 4% and 24%, respectively (*p* = 0.020, *p* = 0.018, respectively). Table S2 presents stratified preferences.

## 4 DISCUSSION

This study explored professionals’ preferences and recommendations for assessment of self‐perceived cognitive functioning in older adults through a global survey. The usage of as many as 16 unique instruments by our sample highlights the ongoing challenge of measurement heterogeneity. Still, the vast majority of respondents indicated a need for a harmonized questionnaire that could identify individuals experiencing cognitive concerns across different settings. There was a broad consensus on several overarching issues, in particular, the need for an instrument to emphasize memory alongside other domains and to assess an individual's current experienced ability as well as decline in that ability over time. A (5‐point) Likert‐scale response format was evidently preferred. Preferences for some questionnaire characteristics and content differed. For example, the top chosen timeframe – the past year – did not reach a clear majority consensus. Likewise, views on most relevant items were varied, and the most popular memory and non‐memory items (“remembering new information” and “attention and concentration”) were each endorsed by about half of respondents. Different preferences for practical features and approaches toward validation also existed within our sample.

Integrating insights from professionals and quantitative statistical information is imperative to enhance an instrument's reliability and content validity and to facilitate acceptability.^18^, ^19^, ^20^, ^21^ IRT modeling approaches provide a solid basis for optimal content validity by identifying potentially relevant items based on information value.^15^, ^22^, ^23^ We observed that the most commonly chosen memory items in our survey assessed specific (future) activities and needing more help to complete activities, the latter being indicative of experienced loss of functional independence. This pattern shows some overlap with the IRT model applied by the SCD‐I Working Group in cognitively unimpaired individuals,^15^ which indicated that memory items that most reliably measured poorer self‐perceived cognitive function pertained to specific activities, such as remembering recent conversations. Interestingly, an earlier study using IRT^23^ showed particular reliability of items that assess global memory function, for example, “Do you think you have problems with your memory?” alongside specific items that assessed problems with, for example, personal dates or phone numbers. We note that this study was based on a mixed sample that included individuals with MCI as well as individuals without formal impairment. The top chosen non‐memory items in our survey were a mix of general (making decisions on everyday matters) and more specific items (handling money). This choice pattern also overlapped with the IRT results, where items that measured most reliably ranged from broad (e.g., focusing on goals or carrying out a plan, making decisions on everyday matters) to specific (trouble recalling the right word). One of our sample's top picks – worry associated with experience decline – is both a key feature of SCD *plus* ^1^ and showed high statistical information value.^13^ Notably, these top chosen items originate from a range of questionnaires, such as the Memory Complaint Questionnaire, Amsterdam Dementia Cohort, subjective cognitive concerns screener, Cognitive Function Index, and Activities of Daily Living Rating Scale Self‐Version, Abbreviated. This indicates that professionals’ preferred items do not stem from one existing instrument and supports the goal of harmonization across instruments.

While there was substantial alignment between the statistical approach and professional perspective, not all statistically informative items were also deemed relevant. These discrepancies between the two sources highlight areas for further investigation and refinement rather than a lack of reliability in one of them. Similar to statistical models that use participant data as input, professionals gather and update their views of the relevance of items through extensive experience with the population. In that sense, their perspective is grounded in a real‐world context: A clinician may, for example, opt to exclude an otherwise informative item because it was difficult to interpret for some individuals without additional clarification by an administrator. Conversely, an item that is not particularly statistically informative may be relevant for an individual's daily life. As IRT models cannot capture such contextual information, the professional opinion adds a deeper understanding of the usefulness of items.

Our respondent sample expressed a broad range of preferences for validation, that is, the use to which an instrument is put.^18^ Top priorities based on choice count were the relationship between self‐perceived cognitive functioning and neuropsychological test performance and the ability to discriminate between cognitive concerns driven by neurodegenerative disorders versus those driven by other factors. Preference for a relationship between self‐perceived cognitive difficulties and test scores may seem counterintuitive given the limited evidence for such a relationship between experienced cognition and traditional test scores,^24^ combined with the fact that SCD excludes the presence of objective impairment.^5^ However, with the conceptualization of AD as a continuum consisting of various stages,^25^ individuals gradually move toward impairment. Early deficits may be found in performance features that are often not the focus of, but can be picked up on with, cognitive testing, such as error types, altered strategies, reduced practice effects, and attenuation of otherwise healthy discrepancies.^26^, ^27^, ^28^, ^29^, ^30^ Digital cognitive testing may help in picking up the subtlest signs of dysfunction.^31^, ^32^ As SCD captures an experienced decline over time, a strong cross‐sectional correlation with performance may be less likely than a longitudinal one. The finding that clinicians were more oriented toward the relationship of an instrument with an individual's daily functioning, while researchers preferred responsiveness of an instrument, seems to reflect the topics these professions deal with on a daily basis.

Various challenges for implementation, including concerns and wishes among user groups, also need to be sufficiently examined early on.^33^ We found that the availability of an informant version and ease of administration were the most desired practical features in our sample, reflecting priorities for the involvement of study partners or informants whose reporting can add value for risk assessment^33^, ^34^ and user‐friendliness. Other features that are crucial to implementation, such as assessment cost,^35^ were less popular. The availability of a normative group and clear administration guidelines were especially popular among clinicians, while researchers more often preferred the possibility of online administration. The reasons behind (different) preferences should be further explored, as self‐perceived cognition is measured across various contexts and settings.

We note several limitations to this study and recommendations for future research. First, although our survey reached professionals from four continents, our input was skewed toward a (Western) European and North American perspective. The relevance of items describing specific activities could differ across cultural and geographical contexts,^36^ potentially limiting the applicability of our results. Second, we preselected items for respondents to choose from, as the entire item bank consisted of over 600 items. Preferences were therefore naturally only gathered within a subgroup of items. Finally, detailed reasons for respondents’ choices are difficult to explore in a survey. To better understand how experts arrive at their decisions regarding item relevance and cultural differences therein, (differences in) their goals of measurement, and potential barriers to implementation, a logical next step would be to conduct focus groups with a representative group of professionals from various cultural, geographical, and professional backgrounds. Together with the SCD‐I Working Group, PIA members can aid in this effort by conducting focus groups in their respective regions. Furthermore, the validation priorities of professionals should simultaneously be put in perspective with statistical results on the relationship between the harmonized Subjective Cognition Factor^15^, ^16^ and different clinical outcomes (e.g., biomarkers, clinical progression, neuropsychological test scores) in cognitively unimpaired older adults. Experts’ priorities can then be weighed against the statistical relationship with the outcomes to further tailor the development of the instrument. A harmonized instrument could ultimately consist of a core set of highly informative items, supplemented with flexible modules to suit additional preferences or measurement goals. Importantly, input from older adults with cognitive concerns and informants should also be gathered in subsequent steps to gather invaluable input about their preferences for practical features and item formats early in the development process. They should also be involved in later instrument testing for usability and user‐friendliness.

In conclusion, professionals who work with SCD populations indicate a need for a harmonized measurement instrument to assess self‐perceived cognitive functioning. Items with both good psychometric qualities and high endorsement by professionals may qualify first. While respondents expressed several clear preferences for how cognitive concerns should be captured, the variability in their recommendations, including practical features and validation, should be further explored. Integrating expert opinion with patient preferences and statistical results can serve as a basis for instrument development.

## CONFLICT OF INTEREST STATEMENT

The authors declare no conflict of interest pertaining to this study. Author disclosures are available in the supporting information.

## CONSENT STATEMENT

All respondents provided informed consent before participating in this study.

## Supporting information



Supporting Information

Supporting Information
