# Supplementary material for: Perspectives on the measurement of self‐perceived cognitive function in older adults
Source: Alzheimers Dement (Amst). 2025 Nov 6;17(4):e70158. doi: 10.1002/dad2.70158 (PMC12591991; doi:10.1002/dad2.70158)
Supplement: Supplementary file 1 — Supporting Information [file DAD2-17-e70158-s001.docx]

**Perspectives on the measurement of self-perceived cognitive function in older adults**

**SUPPLEMENTARY MATERIAL**

| **Supplementary table 1.** Instruments respondents reported using to assess SCD | | |
| --- | --- | --- |
| Abbreviation | Full name | Number of times mentioned |
| ECog Self* | Measurement of Everyday Cognition | 3 |
| CFQ* | Cognitive Failures Questionnaire | 3 |
| BRIEF-A* | Behavior Rating Inventory of Executive Functioning-Adult Version | 3 |
| CCI* | Cognitive Change Index | 2 |
| SCD-I | Subjective Cognitive Decline Interview | 2 |
| CFI* | Cognitive Function Index | 2 |
| CDS-Q* | Cognitive Difficulties Scale | 2 |
| DEX | Dysexecutive Syndrome questionnaire | 2 |
| CLCE-24* | Checklist for Cognitive and Emotional Consequences (after stroke) | 2 |
| 10-item CCQ | 10-item Cognitive Complaint Questionnaire | 1 |
| EMQ | Everyday Memory Questionnaire | 1 |
| FAQ | Functional Activities Questionnaire | 1 |
| MMQ* | Multifactorial Memory Questionnaire | 1 |
| Neuro-QoL | Neurology - Quality of Life | 1 |
| MFQ* | Memory Functioning Questionnaire | 1 |
| TICS-M | Modified Telephone Interview for Cognitive Status | 1 |
| Note: Some respondents mentioned multiple instruments. Some respondents did not mention the names of the instruments they used, despite indicating routinely using an instrument.  * Instrument was also included in the Subjective Cognitive Decline Initiative item bank (Rabin et al., 2023) | | |

A

**Supplementary figure 1.** Selection of memory (A) and non-memory (B) items for researchers and care professionals

B

**Supplementary figure 1.** Selection of memory (A) and non-memory (B) items for researchers and care professionals

| **Supplementary table 2.** Preferences for practical and validation features in a questionnaire as indicated by the experts | | | | | | | | | |  |
| --- | --- | --- | --- | --- | --- | --- | --- | --- | --- | --- |
| **Practical feature** | Not clinician | Clinician | Not researcher | Researcher | **Validation feature** | Not clinician | Clinician | Not researcher | Researcher | |
| Brevity | 14 (30.4%) | 15 (34.1%) | 8 (27.6%) | 21 (34.4%) | Association with CSF biomarkers of dementia | 5 (10.9%) | 1 (2.3%) | 1 (3.4%) | 5 (8.2%) | |
| Cost | 3 (6.5%) | 8 (18.2%) | 2 (6.9%) | 9 (14.8%) | Association with biomarkers of dementia derived from MRI | 5 (10.9%) | 1 (2.3%) | 1 (3.4%) | 5 (8.2%) | |
| Ease of administration (little instruction needed) | 13 (28.3%) | 20 (45.5%) | 11 (37.9%) | 22 (36.1%) | Association with biomarkers derived from PET | 2 (4.3%) | 2 (4.5%) | 0 (0%) | 4 (6.6%) | |
| Possibility to administer online | 17 (37.0%) | 8 (18.2%) | **4 (13.8%)** | **21 (34.4%)** | Association with plasma biomarkers of Alzheimer's disease | 6 (13.0%) | 2 (4.5%) | 3 (10.3%) | 5 (8.2%) | |
| Ease of scoring | 8 (17.4%) | 5 (11.4%) | 4 (13.8%) | 9 (14.8%) | Association with current performance on neuropsychological tests | **17 (37.0%)** | **30 (68.2%)** | 18 (62.1%) | 29 (47.5%) | |
| Has a cut-off score for self-perceived cognitive decline | 10 (21.7%) | 9 (20.5%) | 8 (27.6%) | 11 (18.0%) | Able to dissociate cognitive concerns related to neurodegenerative cognitive decline from concerns related to other factors (e.g., mood, personality, medical variables) | 20 (43.5%) | 27 (61.4%) | 14 (48.3%) | 33 (54.1%) | |
| Covers multiple cognitive domains | 29 (63.0%) | 26 (59.1%) | 11 (37.9%) | 24 (39.3%) | Predictive value for future decline in performance on neuropsychological tests | 17 (37.0%) | 9 (20.5%) | **3 (10.3%)** | **23 (37.7%)** | |
| Clear administration guidelines | **2 (4.3%)** | **9 (20.5%)** | 5 (17.2%) | 6 (9.8%) | Predictive value for future decline in daily functioning (e.g., Instrumental Activities of Daily Living) | **7 (15.2%)** | **19 (43.2%)** | 11 (37.9%) | 15 (24.6%) | |
| Has a companion informant version/form | 18 (39.1%) | 15 (34.1%) | 7 (24.1%) | 26 (42.6%) | Predictive value for clinical diagnosis (of MCI or dementia) | 15 (32.6%) | 20 (45.5%) | 10 (34.5%) | 25 (41.0%) | |
| Availability of an appropriate normative comparison group | **11 (23.9%)** | **21 (47.7%)** | 11 (37.9%) | 21 (34.4%) | Sensitivity to changes in self-perceived cognitive functioning over time (responsiveness) | 16 (34.8%) | 10 (22.7%) | **3 (10.3%)** | **23 (37.7%)** | |
| Other (self-written feature)* | 2 (4.3%) | 1 (2.3%) | 2 (6.9%) | 1 (1.6%) | Other (self-written feature)* | 1 (2.2%) | 0 (0%) | 1 (3.4%) | 0 (0%) | |
| *Choice count = # of times the feature was chosen, Choice % = how much of the total amount of choices made included this feature*  ***Bold*** *= significant difference between groups* | | | | | | | | | |  |
